# Objective Bayesian Inference in Probit Models with Intrinsic Priors Using Variational Approximations

**DOI:** 10.3390/e22050513

**Published:** 2020-04-30

**Authors:** Ang Li, Luis Pericchi, Kun Wang

**Affiliations:** Río Piedras Campus, University of Puerto Rico, 00925 San Juan, Puerto Rico; ang.li@upr.edu (A.L.); kun.wang@upr.edu (K.W.)

**Keywords:** objective Bayesian inference, intrinsic prior, variational inference, binary probit regression, mean-field approximation

## Abstract

There is not much literature on objective Bayesian analysis for binary classification problems, especially for intrinsic prior related methods. On the other hand, variational inference methods have been employed to solve classification problems using probit regression and logistic regression with normal priors. In this article, we propose to apply the variational approximation on probit regression models with intrinsic prior. We review the mean-field variational method and the procedure of developing intrinsic prior for the probit regression model. We then present our work on implementing the variational Bayesian probit regression model using intrinsic prior. Publicly available data from the world’s largest peer-to-peer lending platform, LendingClub, will be used to illustrate how model output uncertainties are addressed through the framework we proposed. With LendingClub data, the target variable is the final status of a loan, either charged-off or fully paid. Investors may very well be interested in how predictive features like FICO, amount financed, income, etc. may affect the final loan status.

## 1. Introduction

There is not much literature on objective Bayesian analysis for binary classification problems, especially for intrinsic prior related methods. By far, only two articles have explored intrinsic prior related methods on classification problems. Reference [1] implements integral priors into the generalized linear models with various link functions. In addition, reference [2] considers intrinsic priors for probit models. On the other hand, variational inference methods have been employed to solve classification problem with logistic regression ([3]) and probit regression ([4,5]) with normal priors. Variational approximation methods have been reviewed in [6,7], and more recently [8].

In this article, we propose to apply variational approximations on probit regression models with intrinsic priors. In Section 4, we review the mean-field variational method that will be used in this article. In Section 3, procedures for developing intrinsic priors for probit models will be introduced following [2]. Our work is presented in Section 5. Our motivations for combining intrinsic prior methodology and variational inference is as following

- Avoiding manually set ad hoc plugin priors by automatically generating a family of non-informative priors that are less sensible.
- Reference [1,2] do not consider inference of posterior distributions of parameters. Their focus is on model comparison. Although the development of intrinsic priors itself comes from a model selection background, we thought it would be interesting to apply intrinsic priors on inference problems. In fact, some recently developed priors that proposed to solve inference or estimation problems turned out to be also intrinsic priors. For example, the Scaled Beta2 prior [9] and the Matrix-*F* prior [10].
- Intrinsic priors concentrate probability near the null hypothesis, a condition that is widely accepted and should be required of a prior for testing a hypothesis.
- Also, intrinsic priors have flat tails that prevents finite sample inconsistency [11].
- For inference problems with large data set, variational approximation methods are much faster than MCMC-based methods.

As for model comparison, due to the fact that the output of variational inference methods cannot be employed directly to compare models, we propose in Section 5.3 to simply make use of the variational approximation of the posterior distribution as an importance function and get the Monte Carlo estimated marginal likelihood by importance sampling for model comparison.

## 2. Background and Development of Intrinsic Prior Methodology

### 2.1. Bayes Factor

The Bayesian framework of model selection coherently involves the use of probability to express all uncertainty in the choice of model, including uncertainty about the unknown parameters of a model. Suppose that models $M_{1},M_{2},...,M_{q}$ are under consideration. We shall assume that the observed data $x=(x_{1},x_{2},...,x_{n})$ is generated from one of these models but we do not know which one it is. We express our uncertainty through prior probability $P(M_{j}),j=1,2,...,q$. Under model $M_{i}$, x has density $f_{i}\left(x|\theta_{i},M_{i}\right)$, where $\theta_{i}$ are unknown model parameters, and the prior distribution for $\theta_{i}$ is $\pi_{i}\left(\theta_{i}|M_{i}\right)$. Given observed data and prior probabilities, we can then evaluate the posterior probability of $M_{i}$ using Bayes’ rule

(1) $$\begin{array}{c} P\left(M_{i}|x\right)=\frac{p_{i}\left(x|M_{i}\right)P\left(M_{i}\right)}{\sum_{j=1}^{q}p_{j}\left(x|M_{j}\right)P\left(M_{j}\right)}, \end{array}$$

where

(2) $$\begin{array}{c} p_{i}\left(x|M_{i}\right)=\int f_{i}\left(x|\theta_{i},M_{i}\right)\pi_{i}\left(\theta_{i}|M_{i}\right)d\theta_{i} \end{array}$$

is the marginal likelihood of x under $M_{i}$, also called the evidence for $M_{i}$ [12]. A common choice of prior model probabilities is $P\left(M_{j}\right)=\frac{1}{q}$, so that each model has the same initial probability. However, there are other alternatives of assigning probabilities to correct for multiple comparison (See [13]). From (1), the posterior odds are therefore the prior odds multiplied by the Bayes factor

(3) $$\begin{array}{c} \frac{P(M_{j}|x)}{P(M_{i}|x)}=\frac{P\left(M_{j}\right)p_{j}\left(x\right)}{P\left(M_{i}\right)p_{i}\left(x\right)}=\frac{P(M_{j})}{P(M_{i})}\times B_{ji}. \end{array}$$

where the Bayes factor of $M_{j}$ to $M_{i}$ is defined by

(4) $$\begin{array}{c} B_{ji}=\frac{p_{j}\left(x\right)}{p_{i}\left(x\right)}=\frac{\int f_{j}\left(x|\theta_{j}\right)\pi_{j}\left(\theta_{j}\right)d\theta_{j}}{\int f_{i}\left(x|\theta_{i}\right)\pi_{i}\left(\theta_{i}\right)d\theta_{i}}. \end{array}$$

Here we omit the dependence on models $M_{j},M_{i}$ to keep the notation simple. The marginal likelihood, $p_{i}\left(x\right)$ expresses the preference shown by the observed data for different models. When $B_{ji}>1$, the data favor $M_{j}$ over $M_{i}$, and when $B_{ji}<1$ the data favor $M_{i}$ over $M_{j}$. A scale for interpretation of $B_{ji}$ is given by [14].

### 2.2. Motivation and Development of Intrinsic Prior

Computing $B_{ji}$ requires specification of $\pi_{i}\left(\theta_{i}\right)$ and $\pi_{j}\left(\theta_{j}\right)$. Often in Bayesian analysis, when prior information is weak, one can use non-informative (or default) priors $\pi_{i}^{N}\left(\theta_{i}\right)$. Common choices for non-informative priors are the uniform prior, $\pi_{i}^{U}\left(\theta_{i}\right)\propto 1$; the Jeffreys prior, $\pi_{i}^{J}\left(\theta_{i}\right)\propto {\left[\det(I_{i}\left(\theta_{i}\right))\right]}^{1/2}$ where $I_{i}\left(\theta_{i}\right)$ is the expected Fisher information matrix corresponding to $M_{i}$.

Using any of the $\pi_{i}^{N}$ in (4) would yield

(5) $$\begin{array}{c} B_{ji}^{N}=\frac{p_{j}^{N}\left(x\right)}{p_{i}^{N}\left(x\right)}=\frac{\int f_{j}\left(x|\theta_{j}\right)\pi_{j}^{N}\left(\theta_{j}\right)d\theta_{j}}{\int f_{i}\left(x|\theta_{i}\right)\pi_{i}^{N}\left(\theta_{i}\right)d\theta_{i}}. \end{array}$$

The difficulty with (5) is that $\pi_{i}^{N}$ are typically improper and hence are defined only up to an unspecified constant $c_{i}$. So $B_{ji}^{N}$ is defined only up to the ratio $c_{j}/c_{i}$ of two unspecified constants.

An attempt to circumvent the ill definition of the Bayes factors for improper non-informative priors is the intrinsic Bayes factor introduced by [15], which is a modification of a partial Bayes factor [16]. To define the intrinsic Bayes factor we consider the set of subsamples x(l) of the data x of minimal size *l* such that $0<p_{i}^{N}\left(x\left(l\right)\right)<\infty$. These subsamples are called training samples (not to be confused with training sample in machine learning). In addition, there is a total number of *L* such subsamples.

The main idea here is that training sample x(l) will be used to convert the improper $\pi_{i}^{N}\left(\theta_{i}\right)$ to proper posterior

(6) $$\begin{array}{c} \pi_{i}^{N}\left(\theta_{i}|x\left(l\right)\right)=\frac{f_{i}\left(x\left(l\right)|\theta_{i}\right)\pi_{i}^{N}\left(\theta_{i}\right)}{p_{i}^{N}\left(x\left(l\right)\right)} \end{array}$$

where $p_{i}^{N}\left(x\left(l\right)\right)=\int f_{i}\left(x\left(l\right)|\theta_{i}\right)\pi_{i}^{N}\left(\theta_{i}\right)d\theta_{i}$. Then, the Bayes factor for the remaining of the data x(n−l), where x(l)∪x(n−l)=x, using $\pi_{i}^{N}\left(\theta_{i}|x\left(l\right)\right)$ as prior is called a “partial” Bayes factor,

(7) $$\begin{array}{c} B_{ji}^{N}\left(x\left(n-l\right)|x\left(l\right)\right)=\frac{\int f_{j}\left(x\left(n-l\right)|\theta_{j}\right)\pi_{j}^{N}\left(\theta_{j}|x\left(l\right)\right)d\theta_{j}}{\int f_{i}\left(x\left(n-l\right)|\theta_{i}\right)\pi_{i}^{N}\left(\theta_{i}|x\left(l\right)\right)d\theta_{i}} \end{array}$$

This partial Bayes factor is a well-defined Bayes factor, and can be written as $B_{ji}^{N}\left(x\left(n-l\right)|x\left(l\right)\right)=B_{ji}^{N}\left(x\right)B_{ij}\left(x\left(l\right)\right)$, where $B_{ji}^{N}\left(x\right)=\frac{p_{j}^{N}\left(x\right)}{p_{i}^{N}\left(x\right)}$ and $B_{ij}\left(x\left(l\right)\right)=\frac{p_{i}^{N}\left(x\left(l\right)\right)}{p_{j}^{N}\left(x\left(l\right)\right)}$. Clearly, $B_{ji}^{N}\left(x\left(n-l\right)|x\left(l\right)\right)$ will depend on the choice of the training samples x(l). To eliminate this arbitrariness and increase stability, reference [15] suggests averaging over all training samples and obtained the arithmetic intrinsic Bayes factor (AIBF)

(8) $$\begin{array}{c} B_{ji}^{AIBF}\left(x\right)=B_{ji}^{N}\left(x\right)\frac{1}{L}\sum_{l=1}^{L}B_{ij}^{N}\left(x\left(l\right)\right). \end{array}$$

The strongest justification of the arithmetic IBF is its asymptotic equivalence with a proper Bayes factor arising from *Intrinsic priors*. These intrinsic priors were identified through an asymptotic analysis (see [15]). For the case where $M_{i}$ is nested in $M_{j}$, it can be shown that the intrinsic priors are given by

(9) $$\begin{array}{c} \pi_{i}^{I}\left(\theta_{i}\right)=\pi_{i}^{N}\left(\theta_{i}\right)\;\mathrm{and}\;\pi_{j}^{I}\left(\theta_{j}\right)=\pi_{j}^{N}\left(\theta_{j}\right)E_{M_{j}}\left[\frac{m_{i}^{N}\left(x\left(l\right)\right)}{m_{j}^{N}\left(x\left(l\right)\right)}|\theta_{j}\right]. \end{array}$$

## 3. Objective Bayesian Probit Regression Models

### 3.1. Bayesian Probit Model and the Use of Auxiliary Variables

Consider a sample $y=(y_{1},...,y_{n})$, where $Y_{i},i=1,...,n$, is a 0−1 random variable such that under model $M_{j}$, it follows a probit regression model with a j+1-dimensional vector of covariates $x_{i}$, where j≤p. Here, *p* is the total number of covariate variables under our consideration. In addition, this probit model $M_{j}$ has the form

(10) $$Y_{i}|\beta_{0},...,\beta_{j},M_{j}\;∼\;\mathrm{Bernoulli}\left(\Phi \left(\beta_{0}x_{0i}+\beta_{1}x_{1i}+...+\beta_{j}x_{ji}\right)\right),\;1\leq i\leq n,$$

where Φ denotes the standard normal cumulative distribution function and $\beta_{j}=\left(\beta_{0},...,\beta_{j}\right)$ is a vector of dimension j+1. The first component of the vector $x_{i}$ is set equal to 1 so that when considering models of the form (10), the intercept is in any submodel. The maximum length of the vector of covariates is p+1. Let π(β), proper or improper, summarize our prior information about β. Then the posterior density of β is given by

$$\begin{array}{c} \pi \left(\beta |y\right)=\frac{\pi \left(\beta \right)\prod_{i=1}^{n}\Phi {\left(x_{i}^{\prime }\beta \right)}^{y_{i}}\left(1-\Phi {\left(x_{i}^{\prime }\beta \right)}^{1-y_{i}}\right)}{\int \pi \left(\beta \right)\prod_{i=1}^{n}\Phi {\left(x_{i}^{\prime }\beta \right)}^{y_{i}}\left(1-\Phi {\left(x_{i}^{\prime }\beta \right)}^{1-y_{i}}\right)d\beta }\;, \end{array}$$

which is largely intractable.

As shown by [17], the Bayesian probit regression model becomes tractable when a particular set of auxiliary variables is introduced. Based on the data augmentation approach [18], introducing *n* latent variables $Z_{1},...,Z_{n}$, where

$$\begin{array}{c} Z_{i}|\beta ∼N\left(x_{i}^{\prime }\beta ,1\right). \end{array}$$

The probit model (10) can be thought of as a regression model with incomplete sampling information by considering that only the sign of $z_{i}$ is observed. More specifically, define $Y_{i}=1$ if $Z_{i}>0$ and $Y_{i}=0$ otherwise. This allows us to write the probability density of $y_{i}$ given $z_{i}$

$$\begin{array}{c} p\left(y_{i}|z_{i}\right)=I\left(z_{i}>0\right)I\left(y_{i}=1\right)+I\left(z_{i}\leq 0\right)I\left(y_{i}=0\right). \end{array}$$

Expansion of the parameter set from {β} to {β,Z} is the key to achieving a tractable solution for variational approximation.

### 3.2. Development of Intrinsic Prior for Probit Models

For the sample $z={\left(z_{1},...,z_{n}\right)}^{\prime }$, the null normal model is

$$M_{1}:\left\{N_{n}\left(z|\alpha 1_{n},I_{n}\right),\pi \left(\alpha \right)\right\}.$$

For a generic model $M_{j}$ with j+1 regressors, the alternative model is

$$M_{j}:\left\{N_{n}\left(z|X_{j}\beta_{j},I_{n}\right),\pi \left(\beta_{j}\right)\right\},$$

where the design matrix $X_{j}$ has dimensions n×(j+1). Intrinsic prior methodology for the linear model was first developed by [19], and was further developed in [20] by using the methods of [21]. This intrinsic methodology gives us an automatic specification of the priors π(α) and π(β), starting with the non-informative priors $\pi^{N}\left(\alpha \right)$ and $\pi^{N}\left(\beta \right)$ for α and β, which are both improper and proportional to 1.

The marginal distributions for the sample z under the null model, and under the alternative model with intrinsic prior, are formally written as

(11) $$\begin{array}{cc} \; & p_{1}\left(z\right)=\int N_{n}\left(z|\alpha 1_{n},I_{n}\right)\pi^{N}\left(\alpha \right)d\alpha ,\\ \; & p_{j}\left(z\right)=\int \int N_{n}\left(z|X_{j}\beta_{j},I_{n}\right)\pi^{I}\left(\beta |\alpha \right)\pi^{N}\left(\alpha \right)d\alpha d\beta . \end{array}$$

However, these are marginals of the sample z, but our selection procedure requires us to compute the Bayes factor of model $M_{j}$ versus the reference model $M_{1}$ for the sample $y=(y_{1},...,y_{n})$. To solve this problem, reference [2] proposed to transform the marginal $p_{j}\left(z\right)$ into the marginal $p_{j}\left(y\right)$ by using the probit transformations $y_{i}=1\left(z_{i}>0\right),i=1,...,n$. These latter marginals are given by

(12) $$\begin{array}{c} p_{j}\left(y\right)=\int_{A_{1}\times ...\times A_{n}}p_{j}\left(z\right)dz \end{array}$$

where

(13) $$\begin{array}{c} A_{i}=\left\{\begin{array}{c} \left(0,\infty \right)\;\mathrm{if}\;y_{i}=1,\\ \left(-\infty ,0\right)\;\mathrm{if}\;y_{i}=0. \end{array}\right. \end{array}$$

## 4. Variational Inference

### 4.1. Overview of Variational Methods

Variational methods have their origins in the 18th century with the work of Euler, Lagrange, and others on the calculus of variations (The derivation in this section is standard in the literature on variational approximation and will at times follow the arguments in [22,23]). Variational inference is a body of deterministic techniques for making approximate inference for parameters in complex statistical models. Variational approximations are a much faster alternative to Markov Chain Monte Carlo (MCMC), especially for large models, and are a richer class of methods than the Laplace approximation [6].

Suppose we have a Bayesian model and a prior distribution for the parameters. The model may also have latent variables, here we shall denote the set of all latent variables and parameters by θ. In addition, we denote the set of all observed variables by X. Given a set of *n* independent, identically distributed data, for which $X=\{x_{1},...,x_{n}\}$ and $\theta =\{\theta_{1},...,\theta_{n}\}$, our probabilistic model (e.g., probit regression model) specifies the joint distribution p(X,θ), and our goal is to find an approximation for the posterior distribution p(θ|X) as well as for the marginal likelihood p(X). For any probability distribution q(θ), we have the following decomposition of the log marginal likelihood

$$\begin{array}{c} \ln p(X)=L(q)+\mathrm{KL}(q||p) \end{array}$$

where we have defined

(14) $$\begin{array}{c} L\left(q\right)=\int q\left(\theta \right)\ln\left\{\frac{p(X,\theta )}{q(\theta )}\right\}d\theta \end{array}$$

(15) $$\begin{array}{c} \mathrm{KL}\left(q||p\right)=-\int q\left(\theta \right)\ln\left\{\frac{p(\theta |X)}{q(\theta )}\right\}d\theta \end{array}$$

We refer to (14) as the lower bound of the log marginal likelihood with respect to the density *q*, and (15) is by definition the Kullback–Leibler divergence of the posterior q(θ|X) from the density *q*. Based on this decomposition, we can maximize the lower bound L(q) by optimization with respect to the distribution q(θ), which is equivalent to minimizing the KL divergence. In addition, the lower bound is attained when the KL divergence is zero, which happens when q(θ) equals the posterior distribution p(θ|X). It would be hard to find such a density since the true posterior distribution is intractable.

### 4.2. Factorized Distributions

The essence of the variational inference approach is approximation to the posterior distribution p(θ|X) by q(θ) for which the *q* dependent lower bound L(q) is more tractable than the original model evidence. In addition, tractability is achieved by restricting *q* to a more manageable class of distributions, and then maximizing L(q) over that class.

Suppose we partition elements of θ into disjoint groups $\left\{\theta_{i}\right\}$ where i=1,...,M. We then assume that the *q* density factorizes with respect to this partition, i.e.,

(16) $$\begin{array}{c} q\left(\theta \right)=\prod_{i=1}^{M}q_{i}\left(\theta_{i}\right). \end{array}$$

The product form is the only assumption we made about the distribution. Restriction (16) is also known as *mean-field* approximation and has its root in Physics [24].

For all distributions q(θ) with the form (16), we need to find the distribution for which the lower bound L(q) is largest. Restriction of *q* to a subclass of product densities like (16) gives rise to explicit solutions for each product component in terms of the others. This fact, in turn, leads to an iterative scheme for obtaining the solutions. To achieve this, we first substitute (16) into (14) and then separate out the dependence on one of the factors $q_{j}\left(\theta_{j}\right)$. Denoting $q_{j}\left(\theta_{j}\right)$ by $q_{j}$ to keep the notation clear, we obtain

(17) $$\begin{array}{c} \begin{array}{cc} L(q) & =\int \prod_{i=1}^{M}q_{i}\left\{\ln p\left(X,\theta \right)-\sum_{i=1}^{M}\ln q_{i}\right\}d\theta \\ \; & =\int q_{j}\left\{\int \ln p\left(X,\theta \right)\prod_{i\neq j}q_{i}d\theta_{i}\right\}d\theta_{j}-\int q_{j}\ln q_{j}d\theta_{j}+\mathrm{constant}\\ \; & =\int q_{j}\ln\tilde{p}\left(X,\theta_{j}\right)d\theta_{j}-\int q_{j}\ln q_{j}d\theta_{j}+\mathrm{constant} \end{array} \end{array}$$

where $\tilde{p}\left(X,\theta_{j}\right)$ is given by

(18) $$\begin{array}{c} \ln\tilde{p}\left(X,\theta_{j}\right)=E_{i\neq j}\left[\ln p\left(X,\theta \right)\right]+\mathrm{constant}. \end{array}$$

The notation $E_{i\neq j}\left[\;\cdot \;\right]$ denotes an expectation with respect to the *q* distributions over all variables $z_{i}$ for i≠j, so that

$$\begin{array}{c} E_{i\neq j}\left[\ln p\left(X,\theta \right)\right]=\int \ln p\left(X,\theta \right)\prod_{i\neq j}q_{i}d\theta_{i}. \end{array}$$

Now suppose we keep the $\left\{q_{i\neq j}\right\}$ fixed and maximize L(q) in (17) with respect to all possible forms for the density $q_{j}\left(\theta_{j}\right)$. By recognizing that (17) is the negative KL divergence between $\tilde{p}\left(X,\theta_{j}\right)$ and $q_{j}\left(\theta_{j}\right)$, we notice that maximizing (17) is equivalent to minimize the KL divergence, and the minimum occurs when $q_{j}\left(\theta_{j}\right)=\tilde{p}\left(X,\theta_{j}\right)$. The optimal $q_{j}^{*}\left(\theta_{j}\right)$ is then

(19) $$\begin{array}{c} \ln q_{j}^{*}\left(\theta_{j}\right)=E_{i\neq j}\left[\ln p\left(X,\theta \right)\right]+\mathrm{constant}. \end{array}$$

The above solution says that the log of the optimal $q_{j}$ is obtained simply by considering the log of the joint distribution of all parameter, latent and observable variables and then taking the expectation with respect to all the other factors $q_{i}$ for i≠j. Normalizing the exponential of (19), we have    

$$\begin{array}{c} q_{j}^{*}\left(\theta_{j}\right)=\frac{\exp(E_{i\neq j}\left[\ln p\left(X,\theta \right)\right])}{\int \exp\left(E_{i\neq j}\left[\ln p\left(X,\theta \right)\right]\right)d\theta_{j}}. \end{array}$$

The set of equations in (19) for j=1,...,M are not an explicit solution because the expression on the right hand side of (19) for the optimal $q_{j}^{*}$ depends on expectations taken with respect to the other factors $q_{i}$ for i≠j. We will need to first initialize all of the factors $q_{i}\left(\theta_{i}\right)$ and then cycle through the factors one by one and replace each in turn with an updated estimate given by the right hand side of (19) evaluated using the current estimates for all of the other factors. Convexity properties can be used to show that convergence to at least local optima is guaranteed [25]. The iterative procedure is described in Algorithm 1.
**Algorithm 1** Iterative procedure for obtaining the optimal densities under factorized density restriction (16). The updates are based on the solutions given by (19).1:Initialize $q_{2}^{*}\left(\theta_{2}\right),\ldots ,q_{M}^{*}\left(\theta_{M}\right).$2:Cycle through
$$\begin{array}{c} q_{1}^{*}\left(\theta_{1}\right)\leftarrow \frac{\exp(E_{i\neq 1}\left[\ln p\left(X,\theta \right)\right])}{\int \exp\left(E_{i\neq 1}\left[\ln p\left(X,\theta \right)\right]\right)d\theta_{1}}\;\\ \vdots \;\\ q_{M}^{*}\left(\theta_{M}\right)\leftarrow \frac{\exp(E_{i\neq M}\left[\ln p\left(X,\theta \right)\right])}{\int \exp\left(E_{i\neq M}\left[\ln p\left(X,\theta \right)\right]\right)d\theta_{M}} \end{array}$$
until the increase in L(q) is negligible.

## 5. Incorporate Intrinsic Prior with Variational Approximation to Bayesian Probit Models

### 5.1. Derivation of Intrinsic Prior to Be Used in Variational Inference

Let $X_{l}$ be the design matrix of a minimal training sample (mTS) of a normal regression model $M_{j}$ for the variable $Z∼N(X_{j}\beta_{j},I_{j+1})$. We have, for the j+1-dimensional parameter $\beta_{j}$,

$$\begin{array}{c} \int N_{j+1}\left(z_{l}|X_{l}\beta_{j},I_{j+1}\right)d\beta_{j}=\left\{\begin{array}{cc} |X_{l}^{\prime }X_{l}{|}^{-1/2} & \mathrm{if}\;\mathrm{rank}\;\mathrm{of}\;X_{l}\geq j+1\\ \infty & \mathrm{otherwise} \end{array}\right.\;. \end{array}$$

Therefore, it follows that the mTS size is j+1 [2]. Given that priors for α and β are proportional to 1, the intrinsic prior for β conditional on α could be derived. Let $\beta_{0}$ denote the vector with the first component equal to α and the others equal to zero. Based on Formula (9), we have

$$\begin{array}{cc} \pi^{I}\left(\beta |\alpha \right) & =\pi_{j}^{N}\left(\beta \right)E_{z_{l}|\beta }^{M_{j}}\left[\frac{p_{1}\left(z_{l}|\alpha \right)}{\int p_{j}\left(z_{l}|\beta \right)\pi_{j}^{N}\left(\beta \right)d\beta }\right]\\ \; & =E_{z_{l}|\beta }^{M_{j}}\left[\frac{\exp\{-\frac{1}{2}{\left(z_{l}-X_{l}\beta_{0}\right)}^{\prime }\left(z_{l}-X_{l}\beta_{0}\right)\}}{\int \exp\{-\frac{1}{2}{\left(z_{l}-X_{l}\beta \right)}^{\prime }\left(z_{l}-X_{l}\beta \right)\}d\beta }\right]\\ \; & ={\left(2\pi \right)}^{-\frac{\left(j+1\right)}{2}}{\left|{\left(X_{l}^{\prime }X_{l}\right)}^{-1}\right|}^{-\frac{1}{2}}\times E_{z_{l}|\beta }^{M_{j}}\left[\exp\{-\frac{1}{2}{\left(z_{l}-X_{l}\beta_{0}\right)}^{\prime }\left(z_{l}-X_{l}\beta_{0}\right)\}\right]\\ \; & ={\left(2\pi \right)}^{-\frac{\left(j+1\right)}{2}}{\left|2{\left(X_{l}^{\prime }X_{l}\right)}^{-1}\right|}^{-\frac{1}{2}}\exp\left\{-\frac{1}{2}\left[{\left(\beta -\beta_{0}\right)}^{\prime }\frac{X_{l}^{\prime }X_{l}}{2}\left(\beta -\beta_{0}\right)\right]\right\}. \end{array}$$

Therefore,

$$\begin{array}{c} \pi^{I}\left(\beta |\alpha \right)=N_{j+1}\left(\beta |\beta_{0},2{\left(X_{l}^{\prime }X_{l}\right)}^{-1}\right),\;\mathrm{where}\;\beta_{0}={\left(\begin{array}{c} \alpha \\ 0\\ \vdots \\ 0 \end{array}\right)}_{(j+1)\times 1}\;. \end{array}$$

Notice that $X_{l}^{\prime }X_{l}$ is unknown because it is a theoretical design matrix corresponding to the training sample $z_{l}$. It can be estimated by averaging over all submatrices containing j+1 rows of the n×(j+1) design matrix $X_{j}$. This average is $\frac{j+1}{n}X_{j}^{\prime }X_{j}$ (See [26] and Appendix A in [2]), and therefore

$$\begin{array}{c} \pi^{I}\left(\beta |\alpha \right)=N_{j+1}\left(\beta |\beta_{0},\frac{2n}{j+1}{\left(X_{j}^{\prime }X_{j}\right)}^{-1}\right). \end{array}$$

Next, based on $\pi^{I}\left(\beta |\alpha \right)$, the intrinsic prior for β can be obtained by

(20) $$\begin{array}{c} \pi^{I}\left(\beta \right)=\int \pi^{I}\left(\beta |\alpha \right)\pi^{I}\left(\alpha \right)d\alpha . \end{array}$$

Since we assume that $\pi^{I}\left(\alpha \right)=\pi^{N}\left(\alpha \right)$ is proportional to one, set $\pi^{N}\left(\alpha \right)=c$ where *c* is an arbitrary positive constant. Denote $\frac{2n}{j+1}{\left(X_{j}^{\prime }X_{j}\right)}^{-1}$ by $\Sigma_{\beta |\alpha }$, we obtain

(21) $$\begin{array}{c} \begin{array}{cc} \pi^{I}\left(\beta \right) & =\int \pi^{I}\left(\beta |\alpha \right)\pi^{I}\left(\alpha \right)d\alpha \\ \; & =c\;\cdot \;{\left(2\pi \right)}^{-\frac{j+1}{2}}{\left|\Sigma_{\beta |\alpha }\right|}^{-\frac{1}{2}}\int \exp\left\{-\frac{1}{2}{\left(\beta -\beta_{0}\right)}^{\prime }\Sigma_{\beta |\alpha }^{-1}\left(\beta -\beta_{0}\right)\right\}d\alpha \\ \; & \propto \exp\left\{-\frac{1}{2}\beta^{\prime }\Sigma_{\beta |\alpha }^{-1}\beta \right\}\times \int \exp\left\{-\frac{1}{2}\left[\beta_{0}^{\prime }\Sigma_{\beta |\alpha }^{-1}\beta_{0}-2\beta^{\prime }\Sigma_{\beta |\alpha }^{-1}\beta_{0}\right]\right\}d\alpha \\ \; & \propto \exp\left\{-\frac{1}{2}\beta^{\prime }\Sigma_{\beta |\alpha }^{-1}\beta \right\}\times \int \exp\left\{-\frac{1}{2}\left(\Sigma_{\beta |\alpha_{\left(1,1\right)}}^{-1}\alpha^{2}-2\beta^{\prime }\Sigma_{\beta |\alpha_{\left(\;\cdot \;1\right)}}^{-1}\alpha \right)\right\}d\alpha \end{array} \end{array}$$

where $\Sigma_{\beta |\alpha_{\left(1,1\right)}}^{-1}$ is component of $\Sigma_{\beta |\alpha }^{-1}$ at position row 1 column 1 and $\Sigma_{\beta |\alpha_{\left(\;\cdot \;1\right)}}^{-1}$ is the first column of $\Sigma_{\beta |\alpha }^{-1}$. Denote $\Sigma_{\beta |\alpha_{\left(1,1\right)}}^{-1}$ by $\sigma_{11}$ and $\Sigma_{\beta |\alpha_{\left(\;\cdot \;1\right)}}^{-1}$ by $\gamma_{1}$, we then obtain

(22) $$\begin{array}{c} \begin{array}{cc} \pi^{I}\left(\beta \right) & \propto \exp\left\{-\frac{1}{2}\beta^{\prime }\Sigma_{\beta |\alpha }^{-1}\beta \right\}\times \int \exp\left\{-\frac{1}{2}\sigma_{11}{\left(\alpha -\frac{\beta^{\prime }\gamma_{1}}{\sigma_{11}}\right)}^{2}+\frac{1}{2}\frac{{(\beta }^{\prime }\gamma_{1}{)}^{2}}{\sigma_{11}}\right\}d\alpha \\ \; & \propto \exp\left\{-\frac{1}{2}\left(\beta^{\prime }\Sigma_{\beta |\alpha }^{-1}\beta -\beta^{\prime }\frac{\gamma_{1}\gamma_{1}^{\prime }}{\sigma_{11}}\beta \right)\right\}\times \sqrt{2\pi }\sigma_{11}^{-1/2}\\ \; & \propto \exp\{-\frac{1}{2}\beta^{\prime }\left(\Sigma_{\beta |\alpha }^{-1}-\frac{\gamma_{1}\gamma_{1}^{\prime }}{\sigma_{11}}\right)\beta \}. \end{array} \end{array}$$

Therefore, we have derived that

(23) $$\begin{array}{c} \pi^{I}\left(\beta \right)\propto N_{j+1}\left(0,{\left(\Sigma_{\beta |\alpha }^{-1}-\frac{\gamma_{1}\gamma_{1}^{\prime }}{\sigma_{11}}\right)}^{-1}\right). \end{array}$$

For model comparison, the specific form of the intrinsic prior may be needed, including the constant factor. Therefore, by following (21) and (22) we have

(24) $$\begin{array}{c} \begin{array}{cc} \pi^{I}\left(\beta \right) & =c\;\cdot \;{\left(2\pi \right)}^{-\frac{j+1}{2}}|\Sigma_{\beta |\alpha }{|}^{-\frac{1}{2}}{\left(2\pi \right)}^{\frac{j+1}{2}}{\left|{\left(\Sigma_{\beta |\alpha }^{-1}-\frac{\gamma_{1}\gamma_{1}^{\prime }}{\sigma_{11}}\right)}^{-1}\right|}^{\frac{1}{2}}\sqrt{2\pi }\sigma_{11}^{-1/2}\times N_{j+1}\left(0,{\left(\Sigma_{\beta |\alpha }^{-1}-\frac{\gamma_{1}\gamma_{1}^{\prime }}{\sigma_{11}}\right)}^{-1}\right)\\ \; & =c\;\cdot \;|\Sigma_{\beta |\alpha }\left(\Sigma_{\beta |\alpha }^{-1}-\frac{\gamma_{1}\gamma_{1}^{\prime }}{\sigma_{11}}\right){|}^{-\frac{1}{2}}\sqrt{2\pi }\sigma_{11}^{-1/2}\times N_{j+1}\left(0,{\left(\Sigma_{\beta |\alpha }^{-1}-\frac{\gamma_{1}\gamma_{1}^{\prime }}{\sigma_{11}}\right)}^{-1}\right)\\ \; & =c\;\cdot \;\sqrt{2\pi }\sigma_{11}^{-1/2}{\left|\left(I-\frac{\gamma_{1}\gamma_{1}^{\prime }}{\sigma_{11}}\Sigma_{\beta |\alpha }\right)\right|}^{-\frac{1}{2}}\times N_{j+1}\left(0,{\left(\Sigma_{\beta |\alpha }^{-1}-\frac{\gamma_{1}\gamma_{1}^{\prime }}{\sigma_{11}}\right)}^{-1}\right). \end{array} \end{array}$$

### 5.2. Variational Inference for Probit Model with Intrinsic Prior

#### 5.2.1. Iterative Updates for Factorized Distributions

We have that

$$\begin{array}{cc} \; & Z_{i}|\beta ∼N\left(x_{i}^{\prime }\beta ,1\right)\;\mathrm{and}\\ \; & p\left(y_{i}|z_{i}\right)=I\left(z_{i}>0\right)I\left(y_{i}=1\right)+I\left(z_{i}\leq 0\right)I\left(y_{i}=0\right) \end{array}$$

in Section 3.1. We have shown in Section 5.1 that

$$\begin{array}{c} \pi^{I}\left(\beta \right)\propto N_{j+1}\left(\mu_{\beta },\Sigma_{\beta }\right), \end{array}$$

where $\mu_{\beta }=0$ and $\Sigma_{\beta }={\left(\Sigma_{\beta |\alpha }^{-1}-\frac{\gamma_{1}\gamma_{1}^{\prime }}{\sigma_{11}}\right)}^{-1}$. Since y is independent of β given z, we have

(25) $$\begin{array}{c} \begin{array}{cc} p(y,z,\beta ) & =p(y|z,\beta )p(z|\beta )p(\beta )\\ \; & =p(y|z)p(z|\beta )p(\beta ). \end{array} \end{array}$$

To apply the variational approximation to probit regression model, unobservable variables are considered in two separate groups, coefficient parameter β and auxiliary variable Z. To approximate the posterior distribution of β, consider the product form

$$\begin{array}{c} q\left(Z,\beta \right)=q_{Z}\left(Z\right)q_{\beta }\left(\beta \right). \end{array}$$

We proceed by first describing the distribution for each factor of the approximation, $q_{Z}\left(Z\right)$ and $q_{\beta }\left(\beta \right)$. Then variational approximation is accomplished by iteratively updating the parameters of each factor distribution.

Start with $q_{Z}\left(Z\right)$, when $y_{i}=1$, we have

$$\begin{array}{cc} \log p(y,z,\beta ) & =\log\left(\prod_{i}\frac{1}{\sqrt{2\pi }}\exp\left\{-\frac{{\left(z_{i}-x_{i}^{\prime }\beta \right)}^{2}}{2}\right\}\times \pi^{I}\left(\beta \right)\right)\;\mathrm{where}\;z_{i}>0. \end{array}$$

Now, according to (19) and Algorithm 1, the optimal $q_{Z}$ is proportional to

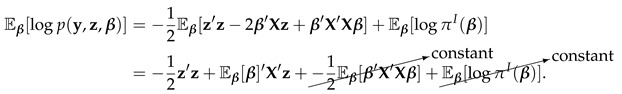


So, we have the optimal $q_{Z}$,

$$\begin{array}{cc} q_{Z}^{*}\left(Z\right) & \propto \exp\{-\frac{1}{2}z^{\prime }z+E_{\beta }{\left[\beta \right]}^{\prime }X^{\prime }z+\mathrm{constant}\}\\ \; & \propto \exp\{-\frac{1}{2}{\left(z-XE_{\beta }\left[\beta \right]\right)}^{\prime }\left(z-XE_{\beta }\left[\beta \right]\right)\}. \end{array}$$

Similar procedure could be used to develop cases when $y_{i}=0$. Therefore, we have that the optimal approximation for $q_{Z}$ is a truncated normal distribution, where

(26) $$\begin{array}{c} q_{Z}^{*}\left(Z\right)=\left\{\begin{array}{c} N_{\left[0,+\infty \right)}\left(XE_{\beta }{\left[\beta \right]}_{i},1\right)\;\mathrm{if}\;y_{i}=1,\\ N_{\left(-\infty ,0\right]}\left(XE_{\beta }{\left[\beta \right]}_{i},1\right)\;\mathrm{if}\;y_{i}=0. \end{array}\right. \end{array}$$

Denote $XE_{\beta }\left[\beta \right]$ by $\mu_{z}$, the location of distribution $q_{Z}^{*}\left(Z\right)$. The expectation $E_{\beta }$ is taken with respect to the density form of q(β) for which we shall derive now.

For $q_{\beta }\left(\beta \right)$, given the joint form in (25), we have

$$\begin{array}{c} \log p\left(y,z,\beta \right)=-\frac{1}{2}\exp\left\{{\left(z-X\beta \right)}^{\prime }\left(z-X\beta \right)\right\}-\frac{1}{2}\exp\left\{{\left(\beta -\mu_{\beta }\right)}^{\prime }\Sigma_{\beta }^{-1}\left(\beta -\mu_{\beta }\right)\right\}+\mathrm{constant}. \end{array}$$

Taking expectation with respect to $q_{Z}\left(z\right)$, we have

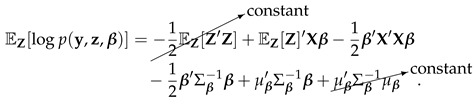


Again, based on (19) and Algorithm 1, the optimal $q_{\beta }\left(\beta \right)$ is proportional to $E_{Z}\left[\log p\left(y,z,\beta \right)\right]$,

$$\begin{array}{c} q_{\beta }^{*}\left(\beta \right)\propto -\frac{1}{2}\beta^{\prime }\left(X^{\prime }X+\Sigma_{\beta }^{-1}\right)\beta +\left(E_{Z}{\left[Z\right]}^{\prime }X+\mu_{\beta }^{\prime }\Sigma_{\beta }^{-1}\right)\beta . \end{array}$$

First notice that any constant terms, including constant factor in the intrinsic prior, were canceled out due to the ratio form of (19). Then by noticing the quadratic form in the above formula we have

(27) $$\begin{array}{c} q_{\beta }^{*}\left(\beta \right)=N\left(\mu_{q_{\beta }},\Sigma_{q_{\beta }}\right), \end{array}$$

where

$$\begin{array}{cc} \Sigma_{q_{\beta }} & ={\left(X^{\prime }X+\Sigma_{\beta }^{-1}\right)}^{-1},\\ \mu_{q_{\beta }} & ={\left(X^{\prime }X+\Sigma_{\beta }^{-1}\right)}^{-1}\left(E_{Z}{\left[Z\right]}^{\prime }X+\mu_{\beta }^{\prime }\Sigma_{\beta }^{-1}\right). \end{array}$$

Notice that $\mu_{q_{\beta }}$, i.e., $E_{\beta }\left[\beta \right]$, depends on $E_{Z}\left[Z\right]$. In addition, from our previous derivation, we found that the update for $E_{Z}\left[Z\right]$ depends on $E_{\beta }\left[\beta \right]$. Given that the density form of $q_{Z}$ is truncated normal, we have

$$\begin{array}{c} E_{Z}\left[Z_{i}\right]=\left\{\begin{array}{cc} XE_{\beta }{\left[\beta \right]}_{i}+\frac{\phi (-XE_{\beta }{\left[\beta \right]}_{i})}{1-\Phi {\left(-XE_{\beta }\left[\beta \right]\right)}_{i}} & \;\mathrm{if}\;y_{i}=1,\\ XE_{\beta }{\left[\beta \right]}_{i}-\frac{\phi (-XE_{\beta }{\left[\beta \right]}_{i})}{\Phi {\left(-XE_{\beta }\left[\beta \right]\right)}_{i}} & \;\mathrm{if}\;y_{i}=0, \end{array}\right. \end{array}$$

where ϕ is the standard normal density and Φ is the standard normal cumulative density. Denote $E_{Z}\left[Z\right]$ by $\mu_{q_{Z}}$. See properties of truncated normal distribution in Appendix A. Updating procedures for parameters $\mu_{q_{\beta }}$ and $\mu_{q_{Z}}$ of each factor distribution are summarized in Algorithm 2.
**Algorithm 2** Iterative procedure for updating parameters to reach optimal factor densities $q_{\beta }^{*}$ and $q_{Z}^{*}$ in Bayesian probit regression model. The updates are based on the solutions given by (26) and (27).1:Initialize $\mu_{q_{Z}}.$2:Cycle through
$$\begin{array}{ccc} \; & \mu_{q_{\beta }}\leftarrow {\left(X^{\prime }X+\Sigma_{\beta }^{-1}\right)}^{-1}\left(\mu_{q_{z}}^{\prime }X+\mu_{\beta }^{\prime }\Sigma_{\beta }^{-1}\right),\; & \mu_{q_{Z}}\leftarrow X\mu_{q_{\beta }}+\frac{\phi (X\mu_{q_{\beta }})}{\Phi {\left(X\mu_{q_{\beta }}\right)}^{y}{\left[\Phi \left(X\mu_{q_{\beta }}\right)-1\right]}^{1-y}}\;, \end{array}$$
until the increase in L(q) is negligible.

#### 5.2.2. Evaluation of the Lower Bound L(q)

During the process of optimization of variational approximation densities, the lower bound for the log marginal likelihood need to be evaluated and monitored to determine when the iterative updating process converges. Based on derivations from previous section, we now have the exact form for the variational inference density,

$$\begin{array}{c} q\left(\beta ,Z\right)=q_{\beta }\left(\beta \right)q_{Z}\left(Z\right). \end{array}$$

According to (14), we can write down the lower bound L(q) with respect to q(β,Z).

(28) $$\begin{array}{c} \begin{array}{cc} L(q) & =\int q\left(\beta ,Z\right)\log\left\{\frac{p(Y,\beta ,Z)}{q(\beta ,Z)}\right\}d\beta dZ\\ \; & =\int q_{\beta }\left(\beta \right)q_{Z}\left(Z\right)\log\left\{\frac{p(Y,\beta ,Z)}{q_{\beta }\left(\beta \right)q_{Z}\left(Z\right)}\right\}d\beta dZ\\ \; & =\int q_{\beta }\left(\beta \right)q_{Z}\left(Z\right)\log\left\{p\left(Y,\beta ,Z\right)\right\}d\beta dZ-\int q_{\beta }\left(\beta \right)q_{Z}\left(Z\right)\log\left\{q_{\beta }\left(\beta \right)q_{Z}\left(Z\right)\right\}d\beta dZ\\ \; & =E_{\beta ,Z}\left[\log\left\{p\left(Y,Z|\beta \right)\right\}\right]+E_{\beta ,Z}\left[\pi^{I}\left(\beta \right)\right]-E_{\beta ,Z}\left[\log\left\{q_{\beta }\left(\beta \right)\right\}\right]-E_{\beta ,Z}\left[\log\left\{q_{Z}\left(Z\right)\right\}\right]. \end{array} \end{array}$$

As we can see in (28), L(q) has been divided into four different parts with expectation taken over the variational approximation density $q\left(\beta ,Z\right)=q_{\beta }\left(\beta \right)q_{Z}\left(Z\right)$. We now find the expression of these expectations one by one.

##### Part 1: $E_{\beta ,Z}\left[\log\left\{p\left(Y,Z|\beta \right)\right\}\right]$

(29) $$\begin{array}{c} \begin{array}{cc} \; & =\log{\left(2\pi \right)}^{-\frac{n}{2}}+\int \int q_{\beta }\left(\beta \right)q_{Z}\left(Z\right)\left\{-\frac{1}{2}{\left(z-X\beta \right)}^{\prime }\left(z-X\beta \right)\right\}d\beta dz\\ \; & =\log{\left(2\pi \right)}^{-\frac{n}{2}}+\int q_{Z}\left(Z\right)\int q_{\beta }\left(\beta \right)\left\{-\frac{1}{2}\left(\beta^{\prime }X^{\prime }X\beta -2z^{\prime }X\beta +z^{\prime }z\right)\right\}d\beta dz \end{array} \end{array}$$

Deal with the inner integral first, we have

(30) $$\begin{array}{c} \begin{array}{cc} \int q_{\beta }\left(\beta \right)\left\{-\frac{1}{2}\left(\beta^{\prime }X^{\prime }X\beta -2z^{\prime }X\beta +z^{\prime }z\right)\right\}d\beta & =-\frac{1}{2}\int q_{\beta }\left(\beta \right)\left[\beta^{\prime }X^{\prime }X\beta \right]d\beta +z^{\prime }XE_{\beta }\left[\beta \right]-\frac{1}{2}z^{\prime }z\\ \; & =-\frac{1}{2}\int q_{\beta }\left(\beta \right)\left[\beta^{\prime }X^{\prime }X\beta \right]d\beta +z^{\prime }X\mu_{q_{\beta }}-\frac{1}{2}z^{\prime }z \end{array} \end{array}$$

where

(31) $$\begin{array}{c} \begin{array}{cc} -\frac{1}{2}\int q_{\beta }\left(\beta \right)\left[\beta^{\prime }X^{\prime }X\beta \right]d\beta & =-\frac{1}{2}\int q_{\beta }\left(\beta \right)\left[{\left(\beta -\mu_{q_{\beta }}+\mu_{q_{\beta }}\right)}^{\prime }X^{\prime }X\left(\beta -\mu_{q_{\beta }}+\mu_{q_{\beta }}\right)\right]d\beta \\ \; & =-\frac{1}{2}\mathrm{trace}\left(X^{\prime }XE_{\beta }\left[\left(\beta -\mu_{q_{\beta }}\right){\left(\beta -\mu_{q_{\beta }}\right)}^{\prime }\right]\right)-\frac{1}{2}\mu_{q_{\beta }}^{\prime }X^{\prime }X\mu_{q_{\beta }}\\ \; & =-\frac{1}{2}\mathrm{trace}\left(X^{\prime }X\left[\mu_{q_{\beta }}\mu_{q_{\beta }}^{\prime }+\Sigma_{q_{\beta }}\right]\right). \end{array} \end{array}$$

Substitute (31) into (30), we got

(32) $$\begin{array}{c} \int q_{\beta }\left(\beta \right)\left\{-\frac{1}{2}\left(\beta^{\prime }X^{\prime }X\beta -2z^{\prime }X\beta +z^{\prime }z\right)\right\}d\beta =-\frac{1}{2}\mathrm{trace}\left(X^{\prime }X\left[\mu_{q_{\beta }}\mu_{q_{\beta }}^{\prime }+\Sigma_{q_{\beta }}\right]\right)+z^{\prime }X\mu_{q_{\beta }}-\frac{1}{2}z^{\prime }z. \end{array}$$

Substituting (32) back into (29) gives

(33) $$\begin{array}{c} \begin{array}{cc} E_{\beta ,Z}\left[\log\left\{p\left(Y,Z|\beta \right)\right\}\right] & =\log{\left(2\pi \right)}^{-\frac{n}{2}}+\int q_{Z}\left(z\right)\left\{-\frac{1}{2}\mathrm{trace}\left(X^{\prime }X\left[\mu_{q_{\beta }}\mu_{q_{\beta }}^{\prime }+\Sigma_{q_{\beta }}\right]\right)+z^{\prime }X\mu_{q_{\beta }}-\frac{1}{2}z^{\prime }z\right\}dz\\ \; & =\log{\left(2\pi \right)}^{-\frac{n}{2}}-\frac{1}{2}\mathrm{trace}\left(X^{\prime }X\left[\mu_{q_{\beta }}\mu_{q_{\beta }}^{\prime }+\Sigma_{q_{\beta }}\right]\right)-\frac{1}{2}E_{Z}\left[z^{\prime }z\right]+\mu_{q_{z}}^{\prime }\mu_{z}\\ \; & =\log{\left(2\pi \right)}^{-\frac{n}{2}}-\frac{1}{2}\mathrm{trace}\left(X^{\prime }X\left[\mu_{q_{\beta }}\mu_{q_{\beta }}^{\prime }+\Sigma_{q_{\beta }}\right]\right)+\mu_{q_{z}}^{\prime }\mu_{z}\\ \; & \;-\frac{1}{2}\sum_{i=1}^{n}{\left[1+\mu_{z_{i}}^{2}-\mu_{z_{i}}\frac{\phi (-\mu_{z_{i}})}{\Phi (-\mu_{z_{i}})}\right]}^{I(y_{i}=0)}{\left[1+\mu_{z_{i}}^{2}+\mu_{z_{i}}\frac{\phi (-\mu_{z_{i}})}{1-\Phi (-\mu_{z_{i}})}\right]}^{I(y_{i}=1)}\\ \; & =\log{\left(2\pi \right)}^{-\frac{n}{2}}-\frac{1}{2}\mathrm{trace}\left(X^{\prime }X\left[\mu_{q_{\beta }}\mu_{q_{\beta }}^{\prime }+\Sigma_{q_{\beta }}\right]\right)+\mu_{q_{z}}^{\prime }\mu_{z}\\ \; & \;-\frac{1}{2}\sum_{i=1}^{n}{\left[1+\mu_{q_{z_{i}}}\mu_{z_{i}}\right]}^{I(y_{i}=0)}{\left[1+\mu_{q_{z_{i}}}\mu_{z_{i}}\right]}^{I(y_{i}=1)}\\ \; & =\log{\left(2\pi \right)}^{-\frac{n}{2}}-\frac{1}{2}\mathrm{trace}\left(X^{\prime }X\left[\mu_{q_{\beta }}\mu_{q_{\beta }}^{\prime }+\Sigma_{q_{\beta }}\right]\right)+\frac{1}{2}\mu_{q_{z}}^{\prime }\mu_{z}-\frac{n}{2}. \end{array} \end{array}$$

We applied properties of truncated normal distribution in Appendix B to find the expression of the second moment $E_{Z}\left[z^{\prime }z\right]$.

##### Part 2: $E_{\beta ,Z}\left[\log q_{Z}\left(z\right)\right]$

(34) $$\begin{array}{c} \begin{array}{cc} \; & =\int \int q_{\beta }\left(\beta \right)q_{Z}\left(z\right)\log q_{Z}\left(z\right)d\beta dZ\\ \; & =\int q_{Z}\left(z\right)\log q_{Z}\left(z\right)dZ\\ \; & =-\frac{n}{2}\left(\log\left(2\pi \right)+1\right)\\ \; & \;+\sum_{i=1}^{n}\left\{{\left[\log\left(\Phi \left(-\mu_{z_{i}}\right)\right)+\mu_{z_{i}}\frac{\phi (-\mu_{z_{i}})}{2\Phi (-\mu_{z_{i}})}\right]}^{I(y_{i}=0)}{\left[\log\left(1-\Phi \left(-\mu_{z_{i}}\right)\right)-\mu_{z_{i}}\frac{\phi (-\mu_{z_{i}})}{2(1-\Phi \left(-\mu_{z_{i}}\right))}\right]}^{I(y_{i}=1)}\right\}\\ \; & =-\frac{n}{2}\left(\log\left(2\pi \right)+1\right)-\frac{1}{2}\mu_{z}^{\prime }\mu_{z}+\frac{1}{2}\mu_{q_{z}}^{\prime }\mu_{z}+\sum_{i=1}^{n}\left\{{\left[\log\left(\Phi \left(-\mu_{z_{i}}\right)\right)\right]}^{I(y_{i}=0)}{\left[\log\left(1-\Phi \left(-\mu_{z_{i}}\right)\right)\right]}^{I(y_{i}=1)}\right\} \end{array} \end{array}$$

Again, see Appendix B for well-known properties of truncated normal distribution. Now subtracting (34) from (33) we got

(35) $$\begin{array}{c} \begin{array}{cc} E_{\beta ,Z}\left[\log\left\{p\left(Y,Z|\beta \right)\right\}\right]-E_{\beta ,Z}\left[\log q_{Z}\left(z\right)\right] & =-\frac{1}{2}\mathrm{trace}\left(X^{\prime }X\left[\mu_{q_{\beta }}\mu_{q_{\beta }}^{\prime }+\Sigma_{q_{\beta }}\right]\right)+\frac{1}{2}\mu_{z}^{\prime }\mu_{z}+\\ \; & \;\sum_{i=1}^{n}\left\{{\left[\log\left(\Phi \left(-\mu_{z_{i}}\right)\right)\right]}^{I(y_{i}=0)}{\left[\log\left(1-\Phi \left(-\mu_{z_{i}}\right)\right)\right]}^{I(y_{i}=1)}\right\}. \end{array} \end{array}$$

Based on the exact expression of the intrinsic prior $\pi^{I}\left(\beta \right)$, denoting all constant terms by *C*, we have

##### Part 3: $E_{\beta ,Z}\left[\log p_{\beta }\left(\beta \right)\right]$

(36) $$\begin{array}{c} \begin{array}{cc} \; & =\int \int q_{Z}\left(z\right)q_{\beta }\left(\beta \right)\log\pi^{I}\left(\beta \right)d\beta dz\\ \; & =\log C-\frac{\left(j+1\right)}{2}\log\left(2\pi \right)-\frac{1}{2}\log\left|\Sigma_{\beta }\right|-\frac{1}{2}\int q_{\beta }\left(\beta \right)\left[\beta^{\prime }\Sigma_{\beta }^{-1}\beta \right]d\beta \end{array} \end{array}$$

To find the expression for the integral, we have

(37) $$\begin{array}{c} \begin{array}{cc} \int q_{\beta }\left(\beta \right)\left[\beta^{\prime }\Sigma_{\beta }^{-1}\beta \right]d\beta & =\int q_{\beta }\left(\beta \right){\left(\beta -\mu_{q_{\beta }}+\mu_{q_{\beta }}\right)}^{\prime }\Sigma_{\beta }^{-1}\left(\beta -\mu_{q_{\beta }}+\mu_{q_{\beta }}\right)d\beta \\ \; & =E\left[\mathrm{trace}\left(\Sigma_{\beta }^{-1}\left(\beta -\mu_{q_{\beta }}\right){\left(\beta -\mu_{q_{\beta }}\right)}^{\prime }\right)\right]+\mu_{q_{\beta }}^{\prime }\Sigma_{\beta }^{-1}\mu_{q_{\beta }}\\ \; & =\mathrm{trace}\left(\Sigma_{\beta }^{-1}\Sigma_{q_{\beta }}\right)+\mu_{q_{\beta }}^{\prime }\Sigma_{\beta }^{-1}\mu_{q_{\beta }} \end{array} \end{array}$$

Substituting (37) back into (36), we obtained

(38) $$\begin{array}{c} E_{\beta ,Z}\left[\log p_{\beta }\left(\beta \right)\right]=\log C-\frac{\left(j+1\right)}{2}\log\left(2\pi \right)-\frac{1}{2}\log\left|\Sigma_{\beta }\right|-\frac{1}{2}\left[\mathrm{trace}\left(\Sigma_{\beta }^{-1}\Sigma_{q_{\beta }}\right)+\mu_{q_{\beta }}^{\prime }\Sigma_{\beta }^{-1}\mu_{q_{\beta }}\right]. \end{array}$$

##### Part 4: $E_{\beta ,Z}\left[\log q_{\beta }\left(\beta \right)\right]$

(39) $$\begin{array}{c} \begin{array}{cc} \; & =\int \int q_{Z}\left(z\right)q_{\beta }\left(\beta \right)\log q_{\beta }\left(\beta \right)d\beta \\ \; & =-\frac{j+1}{2}\log\left(2\pi \right)-\frac{1}{2}\log\left|\Sigma_{q_{\beta }}\right|-\frac{1}{2}\int q_{\beta }\left(\beta \right){\left(\beta -\mu_{q_{\beta }}\right)}^{\prime }\Sigma_{q_{\beta }}^{-1}\left(\beta -\mu_{q_{\beta }}\right)d\beta \\ \; & =-\frac{j+1}{2}\log\left(2\pi \right)-\frac{1}{2}\log\left|\Sigma_{q_{\beta }}\right|-\frac{1}{2}\mathrm{trace}\left(\Sigma_{\beta }^{-1}\Sigma_{\beta }\right)\\ \; & =-\frac{j+1}{2}\left(\log\left(2\pi \right)+1\right)-\frac{1}{2}\log\left|\Sigma_{q_{\beta }}\right| \end{array} \end{array}$$

Combining all four parts together, we get

(40) $$\begin{array}{c} \begin{array}{cc} L(q) & =E_{\beta ,Z}\left[\log\left\{p\left(Y,Z|\beta \right)\right\}\right]+E_{\beta ,Z}\left[\pi^{I}\left(\beta \right)\right]-E_{\beta ,Z}\left[\log\left\{q_{\beta }\left(\beta \right)\right\}\right]-E_{\beta ,Z}\left[\log\left\{q_{Z}\left(Z\right)\right\}\right]\\ \; & =\underset{E_{\beta ,Z}\left[\log\left\{p\left(Y,Z|\beta \right)\right\}\right]-E_{\beta ,Z}\left[\log\left\{q_{Z}\left(Z\right)\right\}\right]}{\underset{︸}{-\frac{1}{2}\mathrm{trace}\left(X^{\prime }X\left[\mu_{q_{\beta }}\mu_{q_{\beta }}^{\prime }+\Sigma_{q_{\beta }}\right]\right)+\frac{1}{2}\mu_{z}^{\prime }\mu_{z}+\sum_{i=1}^{n}\left\{{\left[\log\left(\Phi \left(-\mu_{z_{i}}\right)\right)\right]}^{I(y_{i}=0)}{\left[\log\left(1-\Phi \left(-\mu_{z_{i}}\right)\right)\right]}^{I(y_{i}=1)}\right\}}}\\ \; & \;\underset{E_{\beta ,Z}\left[\log p_{\beta }\left(\beta \right)\right]-E_{\beta ,Z}\left[\log q_{\beta }\left(\beta \right)\right]}{\underset{︸}{+\log C-\frac{1}{2}\log|\Sigma_{\beta }|-\frac{1}{2}\left[\mathrm{trace}\left(\Sigma_{\beta }^{-1}\Sigma_{q_{\beta }}\right)+\mu_{q_{\beta }}^{\prime }\Sigma_{\beta }^{-1}\mu_{q_{\beta }}\right]+\frac{j+1}{2}+\frac{1}{2}\log\left|\Sigma_{q_{\beta }}\right|}}. \end{array} \end{array}$$

### 5.3. Model Comparison Based on Variational Approximation

Suppose we want to compare two models, $M_{1}$ and $M_{0}$, where $M_{0}$ is the simpler model. An intuitive thought on comparing two models by variational approximation methods is just to compare the lower bounds $L(q_{1})$ and $L(q_{0})$. However, we should note that by comparing the lower bounds, we are assuming that the KL divergences in the two approximations are the same, so that we can use just these lower bounds as guide. Unfortunately, it is not easy to measure how tight in theory any particular bound can be, if this can be accomplished we could then more accurately estimate the log marginal likelihood from the beginning. As clarified in [27], when comparing two exact log marginal likelihood, we have

(41) $$\begin{array}{cc} \log p_{1}\left(X\right)-\log p_{0}\left(X\right) & =\left[L\left(q_{1}\right)+KL\left(q_{1}\|p_{1}\right)\right]-\left[L\left(q_{0}\right)-KL\left(q_{0}\|p_{0}\right)\right] \end{array}$$

(42) $$\begin{array}{cc} \; & =L\left(q_{1}\right)-L\left(q_{0}\right)+\left[KL\left(q_{1}\|p_{1}\right)-KL\left(q_{0}\|p_{0}\right)\right] \end{array}$$

(43) $$\begin{array}{cc} \; & \neq L\left(q_{1}\right)-L\left(q_{0}\right). \end{array}$$

The difference in log marginal likelihood, $\log p_{1}\left(X\right)-\log p_{0}\left(X\right)$, is the quantity we wish to estimate. However, if we base this on the lower bounds difference, we are basing our model comparison on () rather than (41). Therefore, there exists a systematic bias towards simpler model when comparing models if $KL\left(q_{1}\|p_{1}\right)-KL\left(q_{0}\|p_{0}\right)$ is not zero.

Realizing that we have a variational approximation for the posterior distribution of β, we propose the following method to estimate p(X) based on our variational approximation $q_{\beta }\left(\beta \right)$ (27). First, writing the marginal likelihood as

$$\begin{array}{c} p\left(x\right)=\int \left[\frac{p\left(x|\beta \right)\pi^{I}\left(\beta \right)}{q_{\beta }\left(\beta \right)}\right]q_{\beta }\left(\beta \right)d\beta , \end{array}$$

we can interpret it as the conditional expectation

$$\begin{array}{c} p\left(x\right)=E\left[\frac{p\left(x|\beta \right)\pi^{I}\left(\beta \right)}{q_{\beta }\left(\beta \right)}\right] \end{array}$$

with respect to $q_{\beta }\left(\beta \right)$. Next, draw samples $\beta^{\left(1\right)},...,\beta^{\left(n\right)}$ from $q_{\beta }\left(\beta \right)$ and obtain the estimated marginal likelihood

$$\begin{array}{c} \hat{p_{X}\left(x\right)}=\frac{1}{n}\sum_{i=1}^{n}\frac{p\left(x|\beta^{\left(i\right)}\right)\pi^{I}\left(\beta^{\left(i\right)}\right)}{q_{\beta }\left(\beta^{\left(i\right)}\right)}. \end{array}$$

Please note that this method proposed is equivalent to importance sampling with importance function being $q_{\beta }\left(\beta \right)$, for which we know the exact form and the generation of the random $\beta^{\left(i\right)}$ is easy and inexpensive.

## 6. Modeling Probability of Default Using Lending Club Data

### 6.1. Introduction

LendingClub (https://www.lendingclub.com/) is the world’s largest peer-to-peer lending platform. LendingClub enables borrowers to create unsecured personal loans between $1000 and $40,000. The standard loan period is three or five years. Investors can search and browse the loan listings on LendingClub website and select loans that they want to invest in based on the information supplied about the borrower, amount of loan, loan grade, and loan purpose. Investors make money from interest. LendingClub makes money by charging borrowers an origination fee and investors a service fee. To attract lenders, LendingClub publishes most of the information available in borrowers’ credit reports as well as information reported by borrowers for almost every loan issued through its website.

### 6.2. Modeling Probability of Default—Target Variable and Predictive Features

Publicly available LendingClub data, from 2007 June to 2018 Q4, has a total of 2,260,668 issued loans. Each loan has a status, either Paid-off, Charged-off, or Ongoing. We only adopted loans with an end status, i.e., either paid-off or charged-off. In addition, that loan status is the target variable. We then selected following loan features as our predictive covariates.

- Loan term in months (either 36 or 60)
- FICO
- Issued loan amount
- DTI (Debt to income ratio, i.e., customer’s total debt divided by income)
- Number of credit lines opened in past 24 months
- Employment length in years
- Annual income
- Home ownership type (own, mortgage, of rent)

We took a sample from the original data set that has customer yearly income between $15,000 and $60,000 and end up with a data set of 520,947 rows.

### 6.3. Addressing Uncertainty of Estimated Probit Model Using Variational Inference with Intrinsic Prior

**Figure 1:**
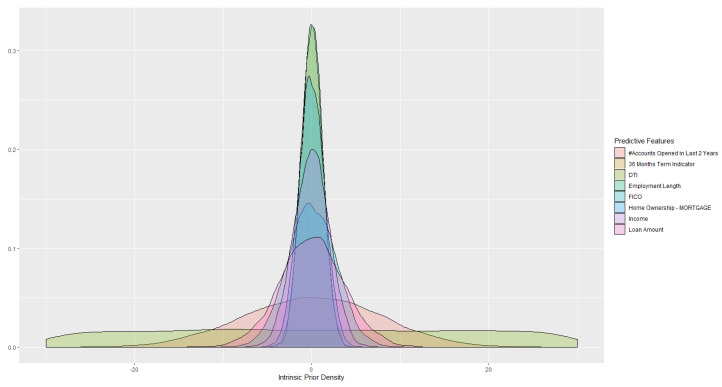
Intrinsic Prior.

Using the process developed in Section 5, we can update the intrinsic prior for parameters (see Figure 1) of the probit model using variational inference, and get the posterior distribution for the estimated parameters. Based on the derived parameter distributions, questions of interest may be explored with model uncertainty being considered.

**Figure 2:**
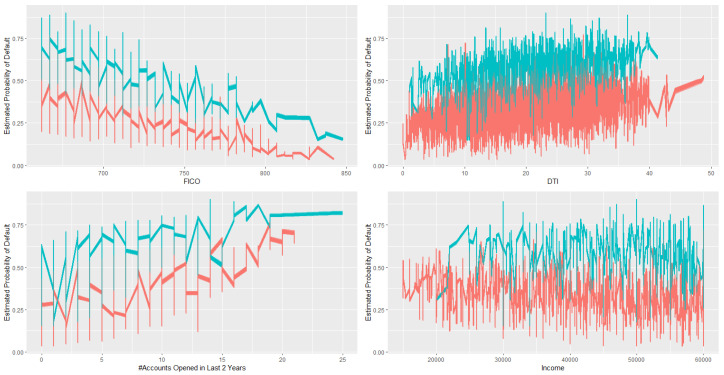
Effect of term months and other covariates on probability of default

Investors will be interested in understanding how each loan feature affect the probability of default, given a certain loan term, either 36 or 60. To answer this question, we samples 6000 cases from the original data set and draw from derived posterior distribution 100 times. We end up with 6000×100 calculated probability of default, where each one of the 6000 samples yield 100 different probit estimates based on 100 different posterior draws. We summarize some of our findings in Figure 2, where color red representing 36 months loans and green representing 60 months loans.

- In general, 60 months loans have higher risk of default.
- Given loan term months, there is a clear trend showing that high FICO means lower risk.
- Given loan term months, there is a trend showing that high DTI indicating higher risk.
- Given loan term months, there is a trend showing that more credit lines opened in past 24 months indicating higher risk.
- There is no clear pattern regarding income. This is probably because we only included customers with income between $15,000 and $60,000 in our training data, which may not representing the true income level of the whole population.

**Figure 3:**
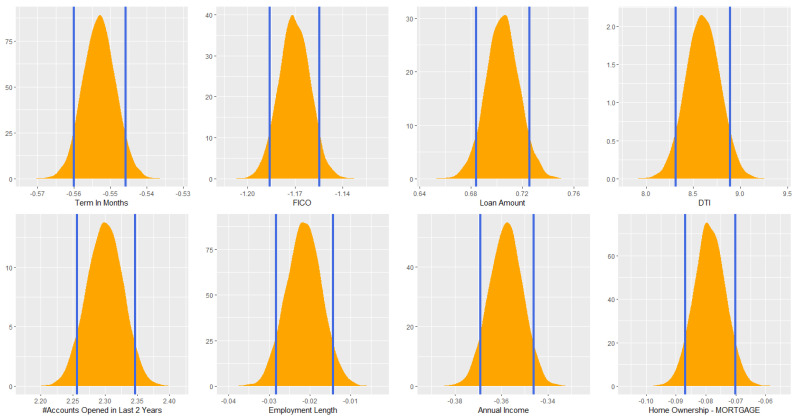
Credible intervals for estimated coefficients

Model uncertainty could also be measured through credible intervals. Again, with the derived posterior distribution, the credible interval is just the range containing a particular percentage of estimated effect/parameter values. For instance, the 95% credible interval of the estimated parameter value of FICO is simply the central portion of the posterior distribution that contains 95% of the estimated values. Contrary to the frequentist confidence intervals, Bayesian credible interval is much more straightforward to interpret. Using the Bayesian framework created in this article, from Figure 3, we can simply state that given the observed data, the estimated effect of DTI on default has 89% probability of falling within [8.300,8.875]. Instead of the conventional 95%, we used 89% following suggestions in [28,29], which is just as arbitrary as any of the conventions.

One of the main advantages of using variational inference over MCMC is that variational inference is much faster. Comparisons were made between the two approximation frameworks on a 64-bit Windows 10 laptop, with 32.0 GB RAM. Using the data set introduced in Section 6.2, we have that

- with a conjugate prior and following the Gibbs sampling scheme proposed by [17], it took 89.86 s to finish 100 simulations for the Gibbs sampler;
- following our method proposed in Section 5.2, it took 58.38 s to get the approximated posterior distribution and sampling 10,000 times from that posterior.

### 6.4. Model Comparison

Following the procedure proposed in Section 5.3, we compare the following series of nested models. From the data set introduced in Section 6.2, 2000 records were sampled to estimate the likelihood $p(x|\beta^{\left(i\right)})$. Where $\beta^{\left(i\right)}$ is one of the 2500 draws sampled directly from the approximated posterior distribution $q_{\beta }\left(\beta \right)$, which serves as the importance function used to estimate the marginal likelihood p(x).

- $M_{2}$: ***FICO*** + ***Term 36 Indicator***
- $M_{3}$: ***FICO*** + ***Term 36 Indicator*** + ***Loan Amount***
- $M_{4}$: ***FICO*** + ***Term 36 Indicator*** + ***Loan Amount*** + ***Annual Income***
- $M_{5}$: ***FICO*** + ***Term 36 Indicator*** + ***Loan Amount*** + ***Annual Income*** + ***Mortgage Indicator***

**Figure 4:**
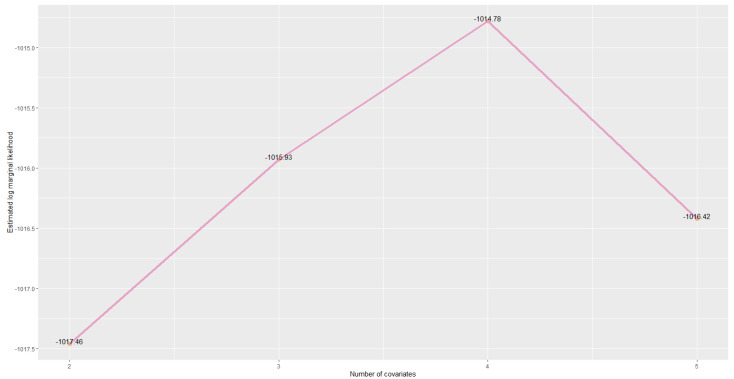
Log marginal likelihood comparison

Estimated log marginal likelihood for each model is plotted in Figure 4. We can see that the model evidence has increased by adding predictive features ***Loan Amount*** and ***Annual Income*** sequentially. However, if we further adding home ownership information, i.e., ***Mortgage Indicator*** as a predictive feature, the model evidence decreased. We have the Bayes factor

$$BF_{45}=\frac{p(x|M_{4})}{p(x|M_{5})}=e^{-1014.78-(-1016.42)}=5.16,$$

which suggests a substantial evidence for model $M_{4}$, indicating home ownership information may be irrelevant in predicting probability of default given that all the other predictive features are relevant.

## 7. Further Work

The authors thank the reviewers for pointing out that mean-field variational Bayes underestimates the posterior variance. This could be an interesting topic for our future research. We plan to study the linearresponsevariationalBayes (LRVB) method proposed in [30] to see if it can be applied on the framework we proposed in this article. To see if we can get the approximated posterior variance close enough to the true variance using our proposed method, comparisons should be made between normal conjugate prior with the MCMC procedure, normal conjugate prior with LRVB, and intrinsic prior with LRVB.

## Appendix A. Density Function

Suppose $X∼N(\mu ,\sigma^{2})$ has a normal distribution and lies within the interval X∈(a,b),−∞≤a<b≤∞. Then *X* conditional on a<X<b has a truncated normal distribution. Its probability density function, *f*, for a≤X<b, is given by

$$\begin{array}{c} f\left(x|\mu ,\sigma ,a,b\right)=\frac{\frac{1}{\sigma }\phi \left(\frac{x-\mu }{\sigma }\right)}{\Phi \left(\frac{b-\mu }{\sigma }\right)-\Phi \left(\frac{a-\mu }{\sigma }\right)} \end{array}$$

and by f=0 otherwise. Here

$$\begin{array}{c} \phi \left(\xi \right)=\frac{1}{\sqrt{2\pi }}\exp\left(-\frac{1}{2}\xi^{2}\right) \end{array}$$

is the probability density function of the standard normal distribution and Φ(·) is its cumulative distribution function. If b=∞, then $\Phi (\frac{b-\mu }{\sigma })=1,$ and similarly, if a=−∞, then $\Phi (\frac{a-\mu }{\sigma })=0.$ And the cumulative density for the truncated normal distribution is

$$\begin{array}{c} F\left(x|\mu ,\sigma ,a,b\right)=\frac{\Phi (\xi )-\Phi (\alpha )}{Z}, \end{array}$$

where $\xi =\frac{x-\mu }{\sigma }$ and Z=Φ(β)−Φ(α).

## Appendix B. Moments and Entropy

Let $\alpha =\frac{a-\mu }{\sigma }$ and $\beta =\frac{b-\mu }{\sigma }$. For two-sided truncation:

$$\begin{array}{cc} \; & E\left(X|a<X<b\right)=\mu +\sigma \frac{\phi (\alpha )-\phi (\beta )}{\Phi (\beta )-\Phi (\alpha )},\\ \; & Var\left(X|a<X<b\right)=\sigma^{2}\left[1+\frac{\alpha \phi (\alpha )-\beta \phi (\beta )}{\Phi (\beta )-\Phi (\alpha )}-{\left(\frac{\phi (\alpha )-\phi (\beta )}{\Phi (\beta )-\Phi (\alpha )}\right)}^{2}\right]. \end{array}$$

For one sided truncation (upper tail):

$$\begin{array}{cc} \; & E(X|X>a)=\mu +\sigma \lambda (\alpha )\\ \; & Var\left(X|X>a\right)=\sigma^{2}\left[1-\delta \left(\alpha \right)\right], \end{array}$$

where $\alpha =\frac{a-\mu }{\sigma },\lambda \left(\alpha \right)=\frac{\phi (\alpha )}{1-\Phi (\alpha )}$ and δ(α)=λ(α)[λ(α)−α].

For one sided truncation (lower tail):

$$\begin{array}{cc} \; & E\left(X|X<b\right)=\mu -\sigma \frac{\phi (\beta )}{\Phi (\beta )}\\ \; & Var\left(X|X<b\right)=\sigma^{2}\left[1-\beta \frac{\phi (\beta )}{\Phi (\beta )}-{\left(\frac{\phi (\beta )}{\Phi (\beta )}\right)}^{2}\right]. \end{array}$$

More generally, the moment generating function for truncated normal distribution is

$$\begin{array}{c} e^{\mu t+\sigma^{2}t^{2}/2}\;\cdot \;\left[\frac{\Phi (\beta -\sigma t)-\Phi (\alpha -\sigma t)}{\Phi (\beta )-\Phi (\alpha )}\right]. \end{array}$$

For a density f(x) defined over a continuous variable, the *entropy* is given by

$$\begin{array}{c} H[x]=-\int f(x)\log f(x)dx. \end{array}$$

And the entropy for a truncated normal density is

$$\begin{array}{c} \log\left(\sqrt{2\pi e}\sigma Z\right)+\frac{\alpha \phi (\alpha )-\beta \phi (\beta )}{2Z}. \end{array}$$
